# Hydralazine Induces Myeloperoxidase and Proteinase 3 Anti-Neutrophil Cytoplasmic Antibody Vasculitis and Leads to Pulmonary Renal Syndrome

**DOI:** 10.1155/2014/868590

**Published:** 2014-08-26

**Authors:** Gaurav Agarwal, Ghayyath Sultan, Sherry L. Werner, Claudia Hura

**Affiliations:** ^1^Division on Nephrology, University of Texas Health Science Center at San Antonio, MC 7882, 7703 Floyd Curl Drive, San Antonio, TX 78229, USA; ^2^Department of Pathology, University of Texas Health Science Center at San Antonio, 7703 Floyd Curl Drive, San Antonio, TX 78229, USA

## Abstract

We report a case of hydralazine-induced ANCA-associated glomerulonephritis with pulmonary hemorrhage. A 62-year-old Hispanic man with hypertension, who was being treated with hydralazine 100 mg three times a day for four and half years, presented to the hospital with severe anemia. He had acute kidney injury and urinalysis showed proteinuria, dysmorphic RBCs, and rare RBC cast. CT scan of the chest revealed bilateral pulmonary ground-glass infiltrates. Transbronchial biopsy was consistent with pulmonary hemorrhage. Serologic tests showed high titer PR3 ANCA and, to a lesser extent, MPO ANCA. Kidney biopsy revealed focal segmental necrotizing glomerulonephritis with crescents, without evidence of immune complex deposits. Hydralazine was discontinued and the patient was treated with corticosteroids and intravenous cyclophosphamide. At one-year follow-up, he had no symptoms and anemia had resolved. Kidney function improved dramatically. Serology showed undetectable PR3 ANCA and minimally elevated MPO ANCA. To our knowledge, hydralazine-associated PR3 ANCA has not been previously reported. The possibility of ANCA systemic vasculitis should be included in the differential diagnosis of any patient with hydralazine use and pulmonary renal syndrome. This is a potentially life threatening condition requiring prompt cessation of the drug and treatment with glucocorticoids and immunosuppression.

## 1. Introduction

Hydralazine was first introduced in 1951 and is widely used as an adjunctive treatment for hypertension [1]. It has been associated with autoimmune diseases. Hydralazine-induced lupus was first reported in 1953 and may be present in as many as 5.4–10.4% of the patients [2]. Occurrence of systemic vasculitis is a rare complication. Drug-induced vasculitis has been associated with hydralazine, propylthiouracil, allopurinol, sulfasalazine, and several other medications [3]. The clinical spectrum can be variable, ranging from arthralgia, myalgia, petechiae, or rash to single- or multiorgan involvement [4]. When it presents as pulmonary renal syndrome, it can have a rapidly progressive course and can be fatal. It is important to have a high index of suspicion, as early diagnosis and treatment can prevent progression of the disease. Discontinuation of drug is usually the first step but many patients subsequently require glucocorticoids and immunosuppressive agents. Here we highlight a rare but severe complication of hydralazine presenting as pulmonary hemorrhage and rapidly progressive glomerulonephritis.

## 2. Case Presentation

A 62-year-old Hispanic man presented to the hospital with generalized weakness, near syncope, and weight loss of 25 lbs over the past four months. He complained of early satiety and lack of appetite. He denied any fever, rash, nasal congestion, myalgia, arthralgia, shortness of breath, cough, hemoptysis, urinary symptoms, or gastrointestinal bleeding. Upon arrival he was found to have severe anemia. He had a history of hypertension, stroke, and hyperlipidemia and was being treated with lisinopril 20 mg daily, metoprolol 100 mg twice a day, aspirin 325 mg daily, simvastatin 40 mg daily, and hydralazine 100 mg three times a day. He had been on hydralazine for the last four and half years. He had no history of renal or lung disease. He quit smoking 20 years ago. Physical exam was unremarkable with stable vital signs. Oxygen saturation was maintained on room air. Upon arrival, he received blood transfusion and his symptoms improved.

**Figure 1:**
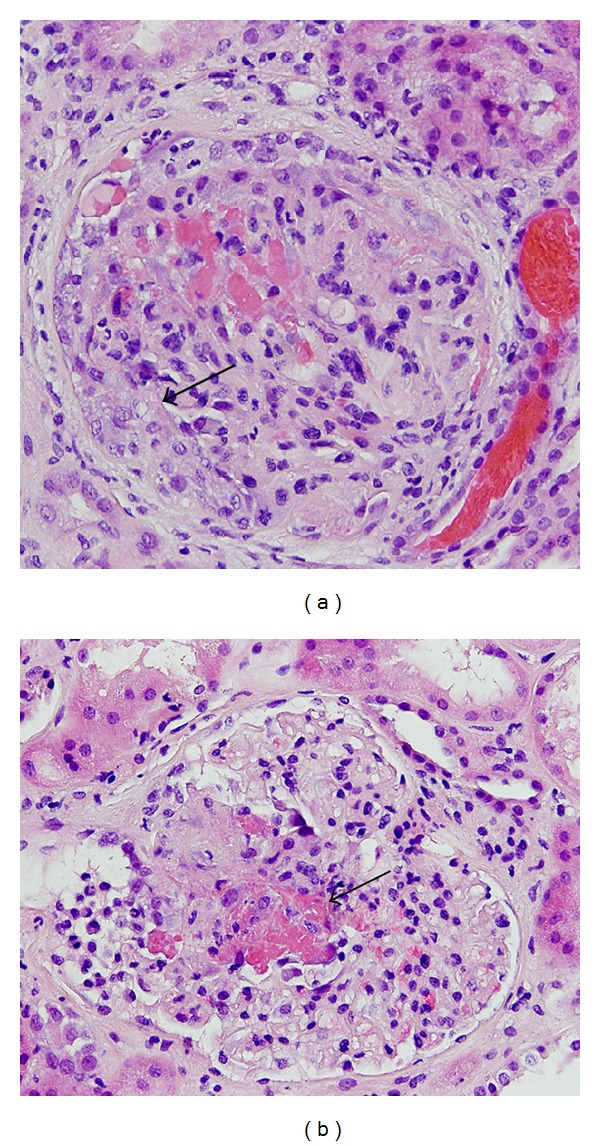
Kidney biopsy. (a) Glomerulus showing cellular crescent (arrow) with proliferation of visceral and parietal epithelial cells (H&E stain). (b) Glomerulus with segmental necrosis with disruption of the basement membrane, nuclear fragments, and fibrin deposition (arrow).

Initial labs showed hemoglobin of 4.1 g/dL, hematocrit of 14.6%, MCV of 64 fl, and platelet count of 557,000/*µ*L. Serum chemistries revealed sodium of 140 mmol/L, potassium of 3.3 mmol/L, chloride of 106 mmol/L, bicarbonate of 24 mmol/L, blood urea nitrogen of 32 mg/dL, and creatinine of 2.13 mg/dL. Urinalysis showed 2+ blood and 2+ protein. He was found to have acute kidney injury with proteinuria of 1742 mg per 24 hours. Examination of the urine sediment showed >50 RBCs per high power field with >50% dysmorphic RBCs and rare RBC cast. Anti-neutrophil cytoplasmic antibody (ANCA) serology showed a predominant increase in proteinase 3 (PR3) titer (55.94 units; normal: 0–21 units) and, to a lesser extent, myeloperoxidase (MPO) titer (23.07 units; normal: 0–21 units). In addition, anti-histone antibody was positive and antinuclear antibody (ANA) titer was elevated at 1 : 640 with a homogenous pattern. Anti-GBM antibody, anti-dsDNA antibody, Smith antibody, SSA, SSB, and ribonucleoprotein antibody were all negative. Hepatitis B surface antigen, hepatitis C antibody, and HIV 1 and 2 antibody were also negative. C3 levels were slightly low (94 mg/dL; normal: 98–162 mg/dL) and C4 levels were within normal range (25 mg/dL). Renal ultrasound was unremarkable. CT scan of the chest revealed bilateral ground-glass infiltrates. Due to unexplained severe anemia, the patient underwent colonoscopy which revealed diverticulosis with one actively bleeding pedunculated polyp in the rectum. There was no evidence of vasculitis involving the colon. EGD revealed blood surrounding the upper esophageal sphincter, which appeared to be emanating from the upper airway. This prompted serial bronchopulmonary lavages and bronchoscopy which were negative for hemorrhage. Subsequently, a transbronchial biopsy was performed and showed intra-alveolar blood and hemosiderin-laden macrophages consistent with pulmonary hemorrhage; there was no evidence of malignancy or granulomas. Kidney biopsy was performed promptly and revealed focal segmental necrotizing glomerulonephritis with 2 of 11 glomeruli showing cellular crescents. Seven glomeruli showed segmental necrosis with disruption of the basement membrane, nuclear fragments, fibrin deposition, a few neutrophils, and obliteration of capillary lumens (Figure 1). Immunofluorescence and electron microscopy were negative for antigen antibody immune complexes. The interstitium showed minimal interstitial fibrosis and tubular atrophy and the vessels were unremarkable.

Hydralazine was discontinued and the patient was treated with three doses of pulse intravenous methylprednisolone 1 gm per dose followed by prednisone 60 mg daily and biweekly taper to 10 mg by 3 months. Concomitantly, he received three monthly pulse doses of intravenous cyclophosphamide at 500 mg per m^2^ of body surface area. The patient was continued on prednisone 5 mg daily for 18 months. His renal function began to improve immediately with resolution of microscopic hematuria. After one month of therapy, MPO and PR3 ANCA became negative and anemia resolved. Six months later, his MPO ANCA became positive with low titer of 27 units. At that time, there was no microscopic hematuria, renal function remained stable, and there was no evidence of recurrent disease. At one-year follow-up, he had no symptoms related to vasculitis and hemoglobin was normal (15.4 g/dL). His kidney function continued to be stable with serum creatinine of 1.1 mg/dL and estimated glomerular filtration rate of 72 mL/min/1.73 m^2^ (MDRD-4). The urine protein creatinine ratio decreased from initial value of 1.7 mg/mg to 0.6 mg/mg. The PR3 ANCA and anti-histone antibody remained undetectable. He continued to have a positive ANA (1 : 160 titer) and low titer MPO ANCA of 32 units. At one and a half years of follow-up, there was no evidence of recurrent renal or pulmonary disease. Overall, this case demonstrates clinicopathologic features consistent with hydralazine-induced pauci-immune ANCA positive glomerulonephritis and provides the first evidence that MPO as well as PR3 ANCAs may be associated with this disease.

## 3. Discussion

ANCA-associated vasculitis is a well-described clinical entity with an incidence of 10–20 cases per million [5]. However, little is known regarding the prevalence of drug-induced ANCA-associated vasculitis (DIV). DIV has been associated with the use of certain drugs including hydralazine, propylthiouracil, minocycline, phenytoin, penicillamine, allopurinol, and sulfasalazine [3]. Although the use of these medications is common, a systemic drug-induced syndrome develops only in a minority of patients. The most frequent symptoms are usually arthralgia, myalgia, and rash. Early withdrawal of the offending drug is usually the only treatment required in the majority of the patients. More advanced disease such as systemic vasculitis could be life threatening and necessitates use of immunosuppressive agents. We report a case of hydralazine-induced pulmonary hemorrhage and pauci-immune crescentic glomerulonephritis with positive MPO, PR3 ANCAs, and anti-histone antibody.

Hydralazine is a direct vasodilator used as an adjuvant treatment for hypertension and heart failure. It is most often implicated as a causal factor in lupus nephritis, with an incidence of 5.4%–10.4%, particularly in patients who are slow acetylators [6]. However, hydralazine-induced ANCA vasculitis is a rare occurrence with pulmonary renal syndrome being the most severe presentation. A review by Yokogawa and Vivino described 37 published cases of hydralazine-induced ANCA-associated vasculitis with kidney involvement [7]. Concomitant occurrence of pulmonary hemorrhage is the most powerful predictor of death [8]. There have been 13 previously reported cases in the literature of hydralazine-induced pulmonary renal syndrome (Table 1). Only 4 out of 10 patients survived. Despite the overall poor prognosis, our patient did well with recovery of renal function and no recurrence of renal or pulmonary disease at one and a half years of follow-up.

The pathogenesis of hydralazine-induced ANCA vasculitis is unknown.* In vivo* data suggests that ANCAs are by themselves pathogenic [9]. MPO knockout mice that lack functioning B- and T-lymphocytes when injected with anti-MPO splenocytes developed severe necrotizing crescentic glomerulonephritis and hemorrhagic pulmonary capillaritis. It has been postulated that hydralazine accumulates in neutrophils where it binds to myeloperoxidase. This induces neutrophil apoptosis and cytotoxic products. The apoptotic blebs of neutrophils act as a source of immunogens as evident by the presence of various antibodies that are associated with hydralazine-induced ANCA vasculitis [10]. These antibodies either alone or by complex interaction with infection agents or genetic factors may contribute to the disease. Antibodies associated with hydralazine-induced vasculitis include MPO ANCA, ANA, anti-histone antibody, anti-elastase antibody, and anti-phospholipid antibody [10, 11]. Surprisingly, our patient was positive for PR3 ANCA in addition to MPO ANCA, ANA, and anti-histone antibody. To our knowledge, the association of hydralazine with PR3 ANCA has not been previously reported. Anti-histone antibody is commonly seen with drug-induced vasculitis and is absent with ANCA-associated vasculitis. The combination of anti-histone antibody, MPO, and/or PR3 ANCA and absence of anti-dsDNA antibody could be used to support the diagnosis of hydralazine-induced vasculitis in the appropriate clinical setting with evidence of pauci-immune glomerulonephritis [12]. A causal role for hydralazine in the pulmonary renal syndrome in the present case is most likely in view of the clinical data, but a definite association would require reexposure to the drug, which is not feasible due to ethical reasons.

In summary, hydralazine-induced ANCA-associated glomerulonephritis with pulmonary hemorrhage is a rare adverse event. Our case demonstrates for the first time that hydralazine may induce both MPO and PR3. Whether coexpression of both ANCAs impacts disease response and/or progression would be of interest. In our case, a short course of immunosuppression with corticosteroids and cyclophosphamide ameliorated the disease. Importantly, our findings indicate that early diagnosis of hydralazine-induced ANCA vasculitis is essential for prompt treatment with cessation of the drug.

## Figures and Tables

**Table 1:** Hydralazine-induced ANCA vasculitis—patients with pulmonary renal syndrome, treatment, and outcome.

Article [reference]	Number of Patients	Positive MPO antibodies	Pulmonary and renal involvement	Treatment of patients with pulmonary renal syndrome	Outcome
Almroth et al. [13]	17	12 of 14 tested	4	C 4/4 Cy 2/4, Az 1/4, P 1/4	3 out of 4 died

Short and Lockwood [14]	10	10	2	C 2/2 Cy 1/2	Not reported

Choi et al. [3]	10	10	5	C 5/5 Cy 5/5	1 out of 4 died

Yokogawa and Vivino [7]	2	2	1	C, Cy	Died

Marina et al. [11]	1	1	1	C, Cy	Died

C: corticosteroids; Cy: cyclophosphamide; Az: azathioprine; P: plasmapheresis.
